# Pyrocarbon humeral head hemiarthroplasty with pectoralis major transfer: a surgical option for young patients with shoulder arthritis and isolated deltoid paralysis: a case report

**DOI:** 10.1016/j.xrrt.2025.07.017

**Published:** 2025-08-13

**Authors:** Tammam Hanna, Diane Ghanem, Evan J. Hernandez, Bassem Elhassan, Brendan MacKay

**Affiliations:** a Department of Orthopaedic Surgery and Rehabilitation, Texas Tech University, TTUHSC - Orthopaedics Department, Lubbock, TX, USA; b Department of Orthopaedic Surgery, The Johns Hopkins Hospital, Baltimore, MD, USA; c Department of Orthopaedic Surgery, Massachusetts General Hospital, Boston, MA, USA

**Keywords:** Deltoid paralysis, Joint preservation, Pectoralis major transfer, Pyrocarbon hemiarthroplasty, Shoulder arthritis, Young adult

The management of shoulder pathology in young, active patients with deltoid atrophy and glenohumeral arthritis in the setting of an intact rotator cuff requires a nuanced approach. While reverse total shoulder arthroplasty with a pectoralis major muscle transfer has become an accepted solution in cases of rotator cuff deficiency, it remains suboptimal in young individuals with osteoarthritis and intact rotator cuff.^4^ The need for an alternative solution prompted the exploration of pyrocarbon as a humeral head replacement option, combined with a pectoralis major rotational flap to restore deltoid function, especially in the settings of intact rotator cuff musculature.^5^^,^^15^^,^^17^

In a 2017 case series, Werthel et al found that patients with complete deltoid paralysis and an intact rotator cuff retain most shoulder functions over time through compensatory mechanisms, including enhanced scapulothoracic motion and increased activation of the clavicular head of the pectoralis major.^19^ However, this functional compensation comes at the expense of heightened mechanical stress on the shoulder, predisposing these patients to early degenerative changes and osteoarthritis due to the absence of deltoid-mediated force distribution.^1^^,^^19^

Pyrocarbon is a biomaterial with exceptional biocompatibility and durability. Originally developed for heart valves, it has gained prominence in orthopedic applications, particularly in small joint arthroplasty, due to its favorable biomechanical properties, including reduced stress shielding, superior wear characteristics, and preservation of native bone compared to cobalt-chrome implants.^5^^,^^6^^,^^10^^,^^13^ European studies have demonstrated that pyrocarbon in shoulder hemiarthroplasty leads to minimal glenoid erosion and maintains satisfactory function at mid-term to long-term follow-up, with reduced revision rates.^3^^,^^4^^,^^6^, ^7^, ^8^^,^^13^ Simultaneously, tendon transfers have been employed to compensate for deltoid and rotator cuff deficiencies, offering improved stability and functional recovery.^16^^,^^18^ The integration of muscle transfer in conjunction with shoulder arthroplasty has been shown to alleviate shoulder pain as well as enhance its control and elevation.^18^

In this case, a 35-year-old male with glenohumeral arthritis post isolated axillary nerve injury underwent pyrocarbon hemiarthroplasty in combination with a pectoralis major pedicled transfer, to address the dual challenge of osteoarthritis and deltoid atrophy. Unlike patients with global brachial plexus injuries, this patient had preserved rotator cuff function, raising the question of whether a muscle transfer was truly necessary. This report aims to clarify the rationale behind this approach and its viability as a reconstructive solution in a young, high-demand patient.

## Case presentation

A male in his mid-30s with a history of a 2012 motorcycle accident presented with progressively worsening left shoulder pain, weakness, and severely restricted motion over the years. The patient had undergone a medial triceps-to-axillary nerve transfer at an outside hospital following his initial injury, which ultimately failed to restore meaningful deltoid function. His weakness in elevating his arm and early fatigability significantly impacted his occupation as a plumber.

**Figure 1:**
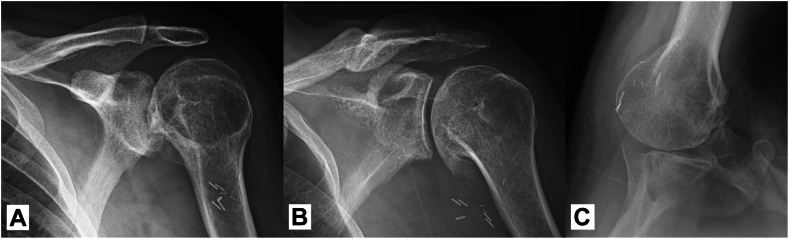
Preoperative X-rays. (**A**) Anteroposterior view demonstrating advanced glenohumeral osteoarthritis with significant degenerative changes. (**B**) Grashey view showing inferior osteophytes and joint space narrowing. (**C**) Axillary view demonstrating posterior subluxation of the humeral head.

**Figure 2:**
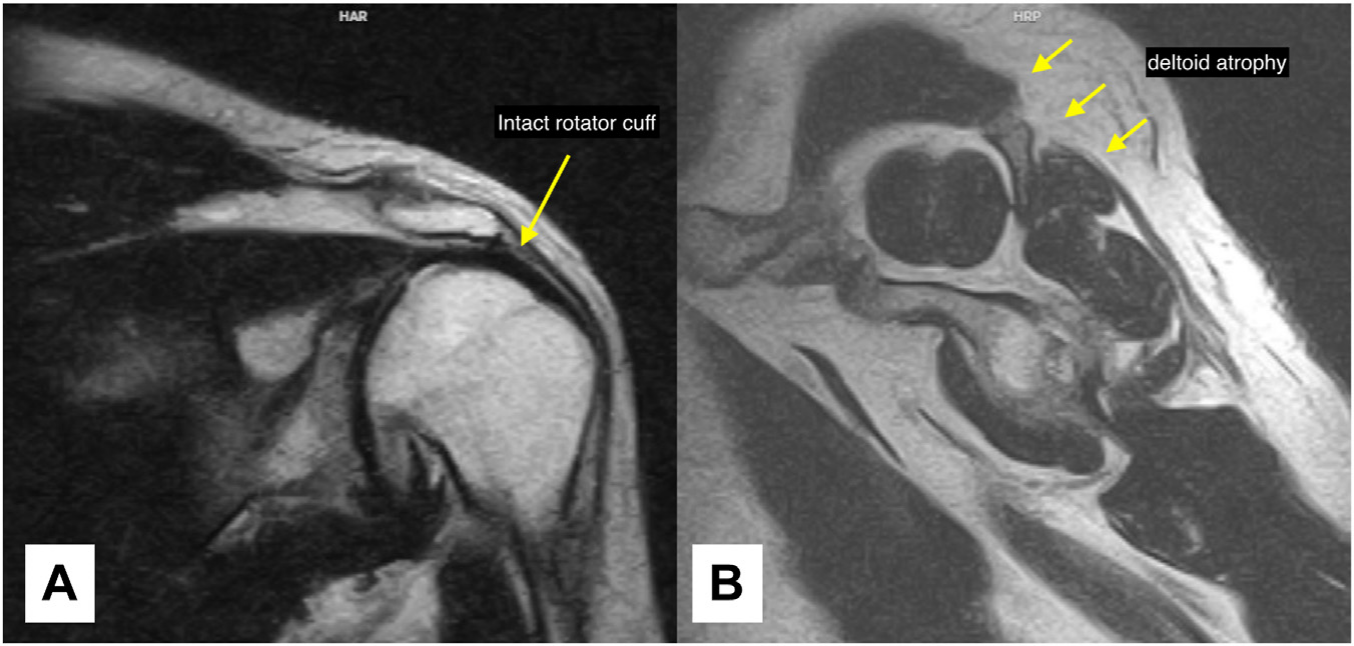
Preoperative MRIs. (**A**) Coronal view demonstrating an intact superior rotator cuff. (**B**) Sagittal view revealing good rotator cuff musculature with evidence of deltoid atrophy. *MRI*, magnetic resonance imaging.

On examination, the patient exhibited profound deltoid atrophy with weak active abduction but preserved rotator cuff function. Bertelli and deltoid extension lag tests were positive.^2^ Radiographic imaging showed posterior subluxation of the humeral head and advanced glenohumeral osteoarthritis with significant degenerative changes (Fig. 1). Magnetic resonance imaging demonstrated intact rotator cuff muscles with evidence of deltoid atrophy (Fig. 2). Given the patient's young age and high functional demands, arthritis, and preserved rotator cuff, a hemiarthroplasty utilizing a pyrocarbon humeral head was chosen, complemented by a pectoralis major pedicled rotational flap to restore deltoid function and preserve native joint structures.

### Surgical Technique

The patient was placed in the beach chair position under general anesthesia. A standard deltopectoral approach was performed, with an extended incision under the clavicle towards the medial sternum in an inverted U shape to facilitate both the arthroplasty and tendon transfer. The cephalic vein was mobilized medially, and the atrophied deltoid was retracted laterally to expose the joint structures. The subscapularis tendon was identified and incised in an L-shaped fashion, tagged with non-absorbable sutures for later repair.

Glenoid preparation involved a ream-and-run technique, using a high-speed burr. Based on preoperative planning and intraoperative trialing, a 48-mm pyrocarbon humeral head was selected and prepared for prosthesis placement, and a size 5B humeral stem was implanted in 30° of retroversion. Fluoroscopy confirmed appropriate implant positioning, and the subscapularis was securely repaired using transosseous sutures.

**Figure 3:**
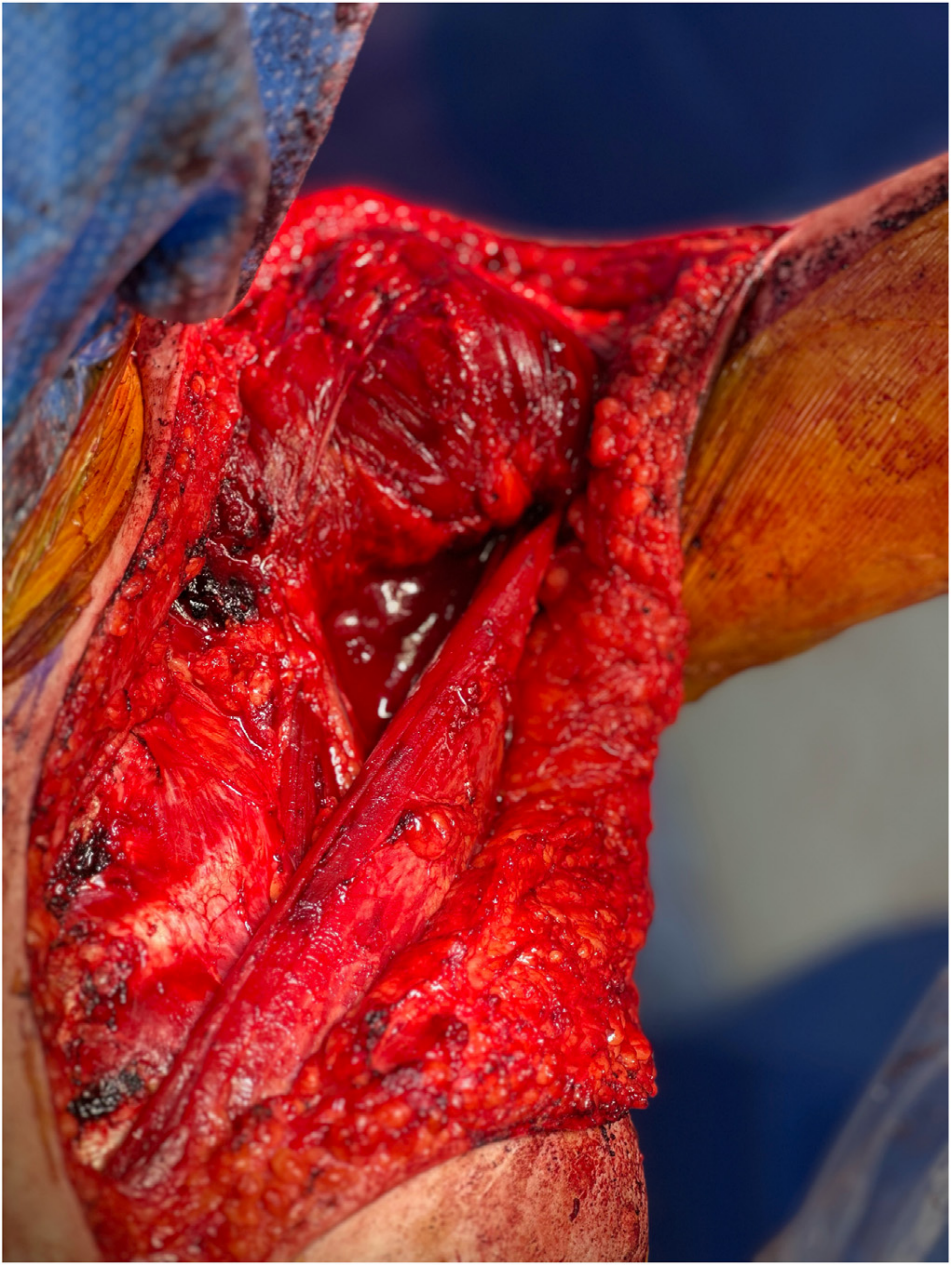
Intraoperative photograph highlighting the preservation of the lower pectoralis major with the upper two-thirds flipped for transfer.

The upper two-thirds of the pectoralis major, including the clavicular and superior sternal heads, were mobilized while preserving the neurovascular pedicle (Fig. 3). Special attention was given to maintaining vascularization and minimizing tension on the pedicle. The muscle was rotated laterally and flipped upside down, effectively repositioning its medial origin laterally to functionally replace the deltoid. To achieve secure fixation, 6 transosseous tunnels were drilled into the lateral clavicle, with an additional 2 tunnels placed in the acromion while maintaining the arm in 80° abduction with 60° forward flexion.

**Figure 4:**
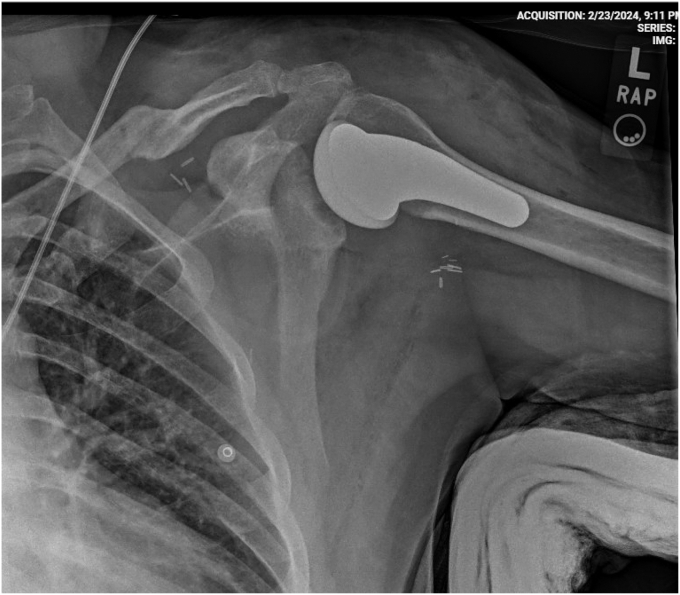
Postoperative Grashey radiograph demonstrating the humeral head position and the application of an abduction spica splint for immobilization.

Hemostasis was carefully maintained throughout the procedure, and a drain was placed before layered closure. A shoulder spica splint was applied to protect the construct and facilitate early healing (Fig. 4).

### Postoperative course and functional outcomes

The patient was maintained in the shoulder spica splint for six weeks to protect the muscle transfer and arthroplasty. Passive range of motion exercises began at six weeks, progressing to active-assisted movements at three months (Table I). Progressive strengthening was introduced at four months, focusing on functional rehabilitation while avoiding direct activation of the pectoralis major (Table I).

**Table I:** Postoperative rehabilitation protocol and return to daily life activities following pyrocarbon hemiarthroplasty with pectoralis major transfer.

Phase	Rehabilitation goals	Allowed activities
Phase 1 (0-6 weeks)	Protect surgical repair, minimize pain, and inflammation	Passive ROM only, no lifting or active shoulder movement, use of sling
Phase 2 (6-12 weeks)	Gradual restoration of passive and active ROM, initiate scapular control exercises	Begin active-assisted ROM, gentle scapular stabilization, no resistance training
Phase 3 (12-16 weeks)	Progressive strengthening, focus on scapular, and rotator cuff muscle activation	Introduce light resistance exercises, continue mobility exercises
Phase 4 (16-24 weeks)	Advanced strengthening, endurance training, begin functional movements	Progressive strengthening with weights, advanced coordination drills
Phase 5 (6 mo+)	Return to work/sports, optimize strength, and coordination	Unrestricted activities, full return to sports/work depending on patient progress

*ROM*, range of motion.

**Figure 5:**
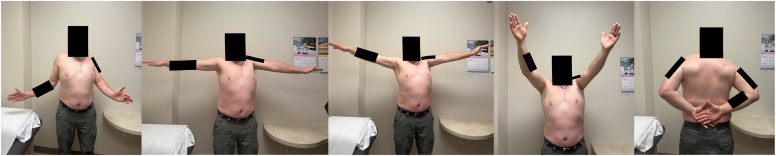
One-year postoperative clinical assessment of range of motion following pyrocarbon hemiarthroplasty and pectoralis major transfer.

**Figure 6:**
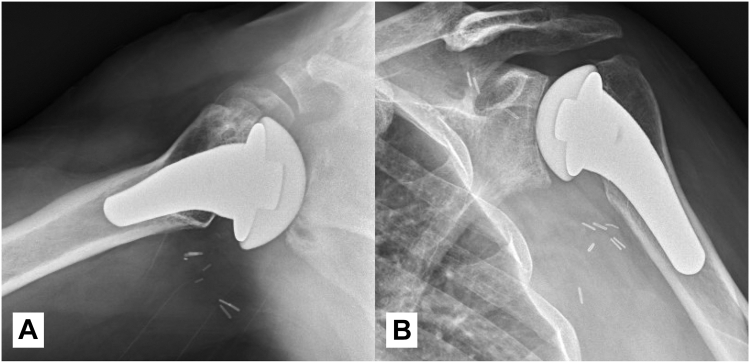
One-year follow-up X-rays. (**A**) Axillary view showing a well-centered humeral head with no evidence of subluxation or loosening. (**B**) Grashey view demonstrating glenoid remodeling with maintained joint congruency.

At 1 year postoperatively, the patient demonstrated forward flexion of 120°, abduction of 110°, external rotation of 20°, and internal rotation to the L4 level (Fig. 5). He reported significant pain relief and was able to return to full duties. Patient-reported outcome measure data showed substantial improvement in the American Shoulder and Elbow Surgeons score, Disabilities of the Arm, Shoulder, and Hand Score, Western Ontario Shoulder Instability Index, and visual analog scale scores (Table II). Radiographic follow-up at 1 year postoperatively confirmed excellent implant alignment, with no evidence of loosening or subsidence (Fig. 6). The pyrocarbon head maintained congruency with the native glenoid (Fig. 6).

**Table II:** Longitudinal assessment of patient-reported outcome measures following pyrocarbon humeral head hemiarthroplasty and pectoralis major rotational flap transfer.

Postoperative time point	VAS∗ (current)	VAS† (worst last 2 Weeks)	VAS‡ (best last 2 Weeks)	ASES§ score	WOSI|| score	DASH¶ score
D 0	20	72	7	33.66	75.3	80
2 weeks	0	50	0	40	65.7	84.2
6 weeks	0	15	0	25	52.1	62.5
12 weeks	0	2	0	78	41	45
4 mo	0	0	0	78	30	20.8
7 mo	0	0	0	85	23	14
10 mo	0	0	0	90	15	10
1 yr	0	0	0	100	3.1	6.7

∗VAS (visual analog scale): A subjective measure of pain intensity, ranging from 0 (no pain) to 100 (worst imaginable pain).

∗ VAS (current): pain level at the time of assessment.

† VAS (worst last 2 weeks): highest reported pain level in the past two weeks.

‡ VAS (best last 2 weeks): lowest reported pain level in the past two weeks.

§ ASES (American Shoulder and Elbow Surgeons score): a standardized outcome measure for shoulder function, combining pain (50%) and functional assessment (50%). The score ranges from 0 to 100, with higher scores indicating better function.

|| WOSI (Western Ontario Shoulder Instability Index): a disease-specific questionnaire assessing quality of life in patients with shoulder instability. The score ranges from 0 (best function) to 100 (worst function), with lower scores representing better outcomes.

¶ DASH (Disabilities of the Arm, Shoulder, and Hand Score): a self-reported questionnaire evaluating upper limb disability and symptoms. The score ranges from 0 (no disability) to 100 (severe disability), with lower scores indicating better function.

## Discussion

Isolated axillary nerve injury is not uncommonly seen in young patients following shoulder trauma, such as dislocations or proximal humerus fractures.^19^ While many recover spontaneously, a subset of patients experience persistent functional deficits despite maintaining an intact rotator cuff.^19^ Werthel et al highlighted that these patients maintain reasonable shoulder function through compensatory mechanisms, including scapulothoracic motion and enhanced activation of the clavicular head of the pectoralis major.^19^ However, this adaptation often comes at the cost of rapid fatigue, progressive pain from joint degeneration, and diminished power, all of which contribute to compromised daily activities and occupational demands.

The management of shoulder dysfunction in young patients with deltoid atrophy, preserved rotator cuff function, and glenohumeral arthritis presents a unique surgical challenge. While reverse total shoulder arthroplasty with pectoralis major transfer is an established treatment option in cases of arthritis with concurrent rotator cuff and deltoid muscle atrophy, concerns surrounding implant longevity, glenoid bone stock, and functional limitations make it a suboptimal choice for young, active individuals with intact cuff musculature.^4^^,^^5^^,^^11^^,^^17^ This case highlights an alternative approach: a pedicled pectoralis flap to compensate for deltoid atrophy and a pyrocarbon hemiarthroplasty to address arthritis, providing a joint-preserving solution for a high-demand patient.

### Pyrocarbon as a novel alternative in shoulder hemiarthroplasty

Pyrocarbon has demonstrated superior biomechanical properties compared to traditional cobalt-chrome implants, and its application in shoulder hemiarthroplasty is slowly increasing.^3^^,^^8^^,^^10^ The decision to use pyrocarbon instead of cobalt-chrome was based on its modulus of elasticity, which closely resembles that of native bone, thereby reducing stress shielding and mitigating subsequent bone loss, a common issue with metallic implants.^7^ In addition, its enhanced wear resistance minimizes glenoid cartilage degradation, making it particularly advantageous for preserving native joint integrity.^5^^,^^10^, ^11^, ^12^ Indeed, in 2018, Klawitter et al demonstrated that pyrocarbon humeral heads cause less bone damage in simulated in vitro tests than cobalt-chrome implants. This characteristic is particularly valuable for young, active patients in whom maintaining glenoid bone stock is essential for future revision options if needed. Garret et al reported significant improvements in pain and function at both short-term and after 5-9 years of follow-up in patients undergoing pyrocarbon hemiarthroplasty for osteoarthritis.^7^^,^^8^ In our case, however, radiographs at 1 year demonstrated calcar resorption. Although pyrocarbon's favorable modulus reduces the risk of stress shielding, the eccentric load transmission from the muscle transfer and altered deltoid vector dynamics may have contributed to this localized remodeling. We have no concrete explanation for this finding, and no conclusions can be drawn regarding implant survivorship under eccentric loading in the setting of muscle transfer. This observation warrants longer-term follow-up and further case accumulation to better understand its clinical significance.

### Pectoralis major pedicled rotational flap: a functional muscle transfer for deltoid deficiency

While studies have shown that patients with deltoid paralysis and an intact rotator cuff retain reasonable shoulder function, they often experience reduced endurance, compensatory scapulothoracic motion, and progressive arthritic changes due to the increased mechanical load on the shoulder.^5^^,^^19^ Some literature has advised against reconstructive interventions in cases of mild symptoms.^9^^,^^14^^,^^19^ However, our patient exhibited debilitating symptoms, significant osteoarthritis, and early fatigability, all of which interfered with his activities of daily living. The increased rotator cuff demand in the absence of the deltoid likely contributed to the patient's arthritis, necessitating an intervention beyond hemiarthroplasty alone.

Addressing deltoid atrophy was a key component of this reconstruction, as traditional hemiarthroplasty relies on an intact muscular envelope for stability. The pectoralis major pedicled rotational flap was chosen due to its ability to restore active shoulder elevation while preserving vascularization and innervation.^11^ Unlike free tendon transfers or static-augmentation procedures, this technique allows for continued neuromuscular function, enabling active contraction and functional overhead movement.^12^ The use of transosseous fixation to the clavicle and acromion provided a biomechanical advantage by improving shoulder stability and optimizing mechanical efficiency for abduction and flexion. Another benefit of this approach is the avoidance of microsurgical complexity, making it an accessible solution for surgeons experienced in shoulder reconstruction. Alternative procedures, such as latissimus dorsi or trapezius transfers, have limitations due to their reliance on rerouted muscle function and the potential for suboptimal tendon attachment, possibly leading to inferior outcomes. In contrast, the repositioning and lateralization of the pectoralis major created a more biomechanically favorable construct, allowing for a more natural and effective restoration of shoulder elevation.

## Conclusion

This case demonstrates the successful integration of pyrocarbon hemiarthroplasty and pectoralis major transfer as a viable approach for young patients with isolated deltoid atrophy and glenohumeral arthritis The early functional outcomes have been promising, showing pain relief, improved shoulder elevation, and no evidence of implant loosening or migration.

Although further research is needed to evaluate the long-term durability of this approach, the combination of pyrocarbon hemiarthroplasty and functional restoration via pectoralis major transfer offers a potential treatment option for patients with deltoid atrophy, intact rotator cuff function, and progressive osteoarthritis.

## Disclaimers

Funding: No funding was disclosed by the authors.

Conflicts of interest: The authors, their immediate families, and any research foundation with which they are affiliated have not received any financial payments or other benefits from any commercial entity related to the subject of this article.

Patient consent: Obtained.
